# Anterior Cord Syndrome Due to Spontaneous Spinal Epidural Hematoma

**DOI:** 10.7759/cureus.62149

**Published:** 2024-06-11

**Authors:** Erin G Park, Sarah Seghrouchni, Rita K Bliesner, Maria Carmona-Gonzalez

**Affiliations:** 1 Medicine, Alabama College of Osteopathic Medicine, Dothan, USA; 2 Internal Medicine, Ascension Sacred Heart Pensacola, Pensacola, USA

**Keywords:** spontaneous spinal epidural hematoma, acs and sseh, sseh unknown etiology, sseh, anterior cord syndrome

## Abstract

Spontaneous spinal epidural hematoma (SSEH) represents a rare clinical entity with an indeterminate etiology. Timely diagnosis and intervention are imperative due to the significant risk of permanent neurological deficits in the absence of appropriate treatment. This case report presents an instance of SSEH with no clear etiology. The patient arrived at the emergency department with paraplegia, urinary and fecal incontinence, and loss of pain and temperature sensation. She reported that these symptoms began abruptly after sneezing. The patient denied any pertinent medical history or family history. The patient initially experienced epigastric pain, which progressed to paresthesia. Magnetic resonance imaging confirmed an epidural hematoma extending from T2 to T8, necessitating immediate neurosurgical intervention. Although the patient was expected to recover within 72 hours postoperation, her symptoms persisted. Based on her clinical presentation, a diagnosis of anterior cord syndrome secondary to SSEH was confirmed.

## Introduction

Anterior cord syndrome (ACS) stands as an intricate manifestation of spinal cord injury, often arising from traumatic or vascular insults to the anterior spinal artery. In the realm of neurosurgery, its occurrence is infrequent with less than 1% of all spinal cord injuries being ACS [1], yet its impact on patients can be profound, leading to a spectrum of motor, sensory, and autonomic disturbances. In the context of spontaneous spinal epidural hematoma (SSEH), ACS emerges as a poignant example of the complexities inherent in neurological emergencies. SSEH represents a clinical rarity, characterized by the sudden accumulation of blood within the epidural space of the spinal cord, precipitating acute neurological deficits [2]. While the exact cause of SSEH remains unclear, potential explanations include arteriovenous malformations, the rupture of epidural vessels, or issues with epidural veins [3]. Surgical intervention is promptly required to evacuate the hematoma and relieve the compression on the spinal cord [4].

Specifically to our case report, the convergence of SSEH and ACS represents an exceedingly rare occurrence, adding layers of complexity to an already intricate medical landscape. Despite thorough examination, including coagulation tests, the core reason behind this tragic event remains elusive, highlighting the mysterious complexity inherent in neurological emergencies. This perplexing scenario underscores the urgent need for further research and heightened vigilance in the realm of neurosurgery, where even the most uncommon occurrences can have profound and life-altering consequences for patients like ours.

In this case report, we present a 32-year-old female, whose initial presentation of numbness around the epigastric area swiftly progressed to paraplegia and paresthesia, SSEH serves as a stark reminder of the swift and devastating consequences such pathologies can wield. We aim to explore the intricate interplay between SSEH and ACS, shedding light on their clinical manifestations and management strategies.

## Case presentation

A 32-year-old female with no significant past medical history presented to the emergency department with the complaint of diffuse abdominal pain, which progressed to descending paresthesia within a 24-hour period. The patient recollected sneezing while grocery shopping, and feeling pain in her epigastric area. She did not go to the emergency room until the pain progressed to paresthesia. Upon physical exam, the patient presented with diminished pain and temperature sensation in the bilateral lower extremities. The patient was able to distinguish soft touch on her lower extremities but was not able to follow commands to move her lower extremities. The exam also showed a ⅕ lower extremity muscle weakness and no patellar reflex. These physical exam findings confirmed the diagnosis of ACS.

**Figure 1:**
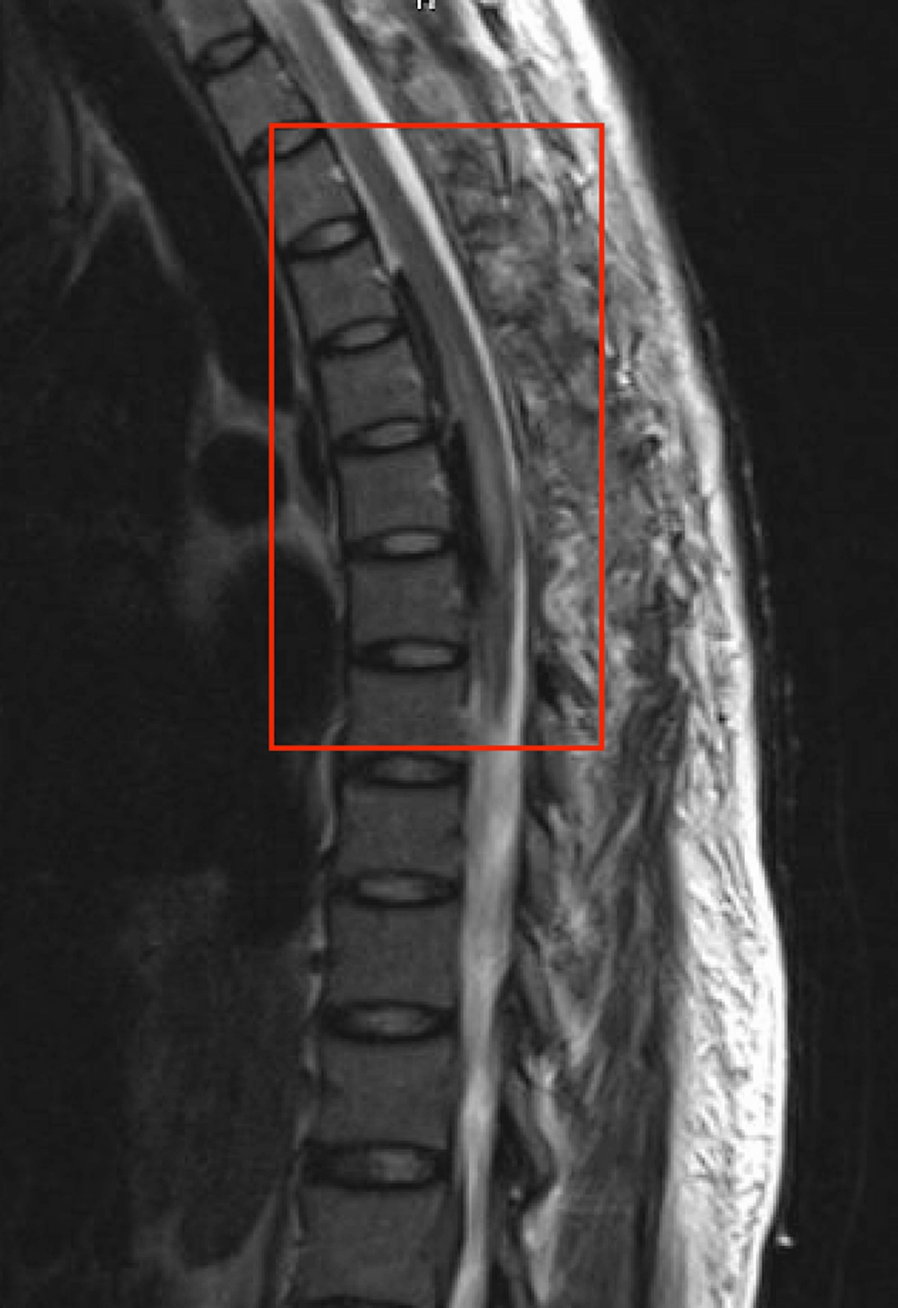
T1-weighted MRI of the thoracic spine portrays the epidural hematoma that ranged from T2 to T8.

**Figure 2:**
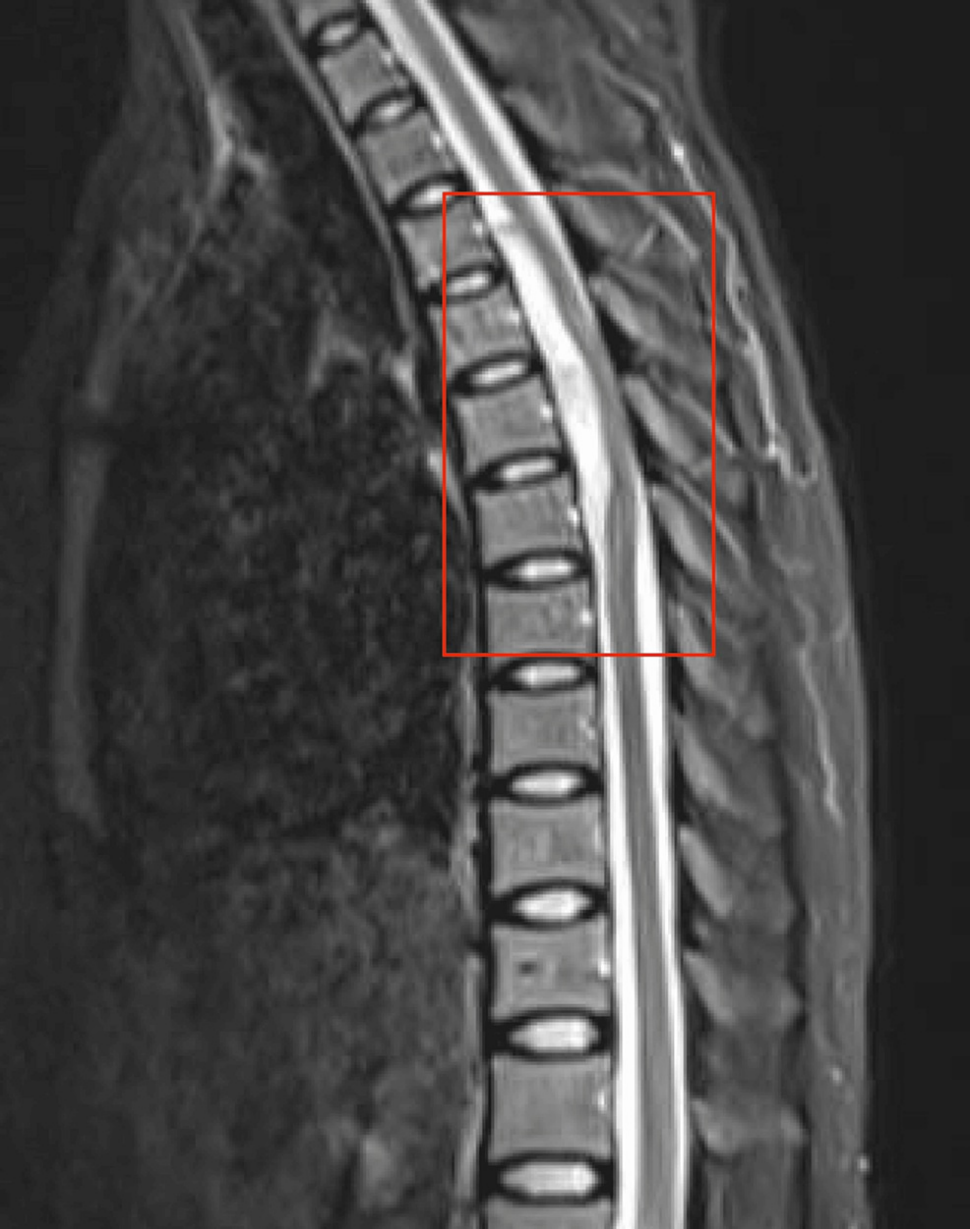
T2-weighted MRI of the thoracic spine portrays the epidural hematoma that ranged from T2 to T8.

Magnetic resonance imaging (MRI) confirmed the diagnosis of an epidural hematoma from T2 to T8 (Figures 1, 2). Under general anesthesia, thoracic and medial laminectomy was performed. However, three days after the procedure, the patient showed persistent paraplegia and urinary and fecal incontinence. A spinal angiogram was ordered and showed no evidence of a current bleed. Coagulation studies did not show a vascular etiology. The patient denied pertinent family history.

The patient was ultimately discharged with orders to continue intense physical and occupational therapy (PT/OT) at home. The patient was also referred to hematology to find the underlying cause of the SSEH and to manage possible anticoagulants. After months of continued PT/OT, the patient reports improved motor function and is able to support her gait with a walker. She no longer requires a urinary catheter. However, she does note radiculopathy and a burning sensation in her bilateral lower extremities with each therapy session.

## Discussion

SSEH is a rare idiopathic neurosurgical emergency resulting from blood accumulation in the vertebral epidural space. The etiology is still currently unknown, but predisposing factors include vascular malformations, pregnancy, and coagulation dysfunctions [5,6]. Our patient presented with none of these known predisposing risks. The incidence of SSEH is estimated to be 0.1 patients per 100,000 per year [7]. Some literature has shown that spinal epidural hematomas present more commonly in the dorsal space due to the anatomy of the vertebral foramen and more extensive posterior venous plexus [5]. SSEH can present as a range of symptoms from back pain to paraplegia depending on the spinal level affected. From the literature review, we found only a handful of cases that presented similarly to our patient. A posterior SSEH between the C3 and T5 levels led to progressive tetraparesis and a loss of superficial body sensation below the C8 level, while deep sensation remained entirely unaffected [8]. However, this patient had a history of disc herniation at the C6-C7 level while our patient had no pertinent medical history.

ACS typically presents with acute onset back or neck pain leading to bilateral sensory and motor loss and autonomic dysfunction [9]. These symptoms are due to the disruption in the vascular supply of the anterior spinal artery found on the anterior midline of the spinal cord. Obstruction of the anterior spinal artery leads to lesions of the corticospinal tracts involving the motor relay system and the spinothalamic tracts involving pain, temperature, and sensation [9]. A key clinical feature of ACS is the retention of vibratory and position sense below the level of the lesion. Involvement of the lateral horns in T1-L2 causes autonomic symptoms, including a neurogenic bladder or bowel, hypotension, and sexual dysfunction [9]. The patient in this case presented with fecal and urinary incontinence, diminished pain and temperature sensation, and numbness of the epigastric area progressing to paraplegia and paresthesia, which support a diagnosis of ACS. According to previous literature, another case of a ventral SSEH causing ACS in an adult was reported in 1981 at the University of Michigan [10]. In 2015, a ventral SSEH was reported in South Korea, but the patient presented with paraplegia and loss of proprioception while retaining touch sensation [11].

Rapid clinical assessment and imaging are required to minimize deficits stemming from the spinal epidural hematoma. MRI should be performed if there is high clinical suspicion of ACS to rule out disc herniation, spinal abscess or mass, traumatic injury, and hemorrhagic stroke. With prompt diagnosis and management, many patients presenting with SSEH are able to achieve significant neurological recovery. Some investigators have reported that neurological improvement occurred in the first 24 hours in 63.2% of SSEH cases and complete neurological recovery was attained within one month in 78.9% of cases [12]. Additionally, the hematoma resolved in the first month for 73.7% of cases according to radiological images [12]. With only one previously reported case of ACS due to SSEH and our patient having an unknown etiology, treatment focused on spinal decompression to alleviate her acute symptoms and prevent further neurological damage. Initial surgical intervention for SSEH typically consists of laminectomy to reduce spinal cord retraction and encroachment. However, with minimal prior literature and unknown etiologies, long-term management of patients with SSEH can be complicated. This case was exceptionally difficult due to the ventral position of the SSEH and the lack of past medical history to guide treatment. There have only been two reported cases of recurrent SSEH, one in 1982 in Ontario and one in 1997 in Taiwan [13,14]. Therefore, there is a low risk of this recurring in the patient, but due to the spontaneous nature of the condition, it is not something that can be dismissed. Overall, SSEH is difficult to treat and manage, but immediate surgical intervention is key to maximize recovery and maintain the patient’s quality of life.

## Conclusions

The etiology of SSEH in this patient remains unknown. However, prompt neurosurgical intervention, followed by intensive physical and occupational therapy, enabled the patient to regain most of her function. Due to the rarity of SSEH, there are not enough reported cases to fully understand the optimal course of treatment. Nevertheless, all documented cases emphasize the importance of prompt neurosurgical intervention.
